# Endolymphatic hydrops asymmetry distinguishes patients with Meniere’s disease from normal controls with high sensitivity and specificity

**DOI:** 10.3389/fneur.2023.1280616

**Published:** 2023-12-21

**Authors:** Tae-Soo Noh, Moo Kyun Park, Jun Ho Lee, Seung Ha Oh, Ji-hoon Kim, In Chan Song, Myung-Whan Suh

**Affiliations:** ^1^Department of Otorhinolaryngology-Head and Neck Surgery, Seoul National University College of Medicine, Seoul National University Hospital, Seoul, Republic of Korea; ^2^Department of Radiology, Seoul National University College of Medicine, Seoul National University Hospital, Seoul, Republic of Korea

**Keywords:** endolymphatic Hydrops, Meniere’s disease, sensitivity, specificity, vestibule

## Abstract

**Background:**

Many endolymphatic hydrops (EH) MRI studies in the literature do not include a normal control group. Consequently, it remains unclear which outcome measure in EH MRI can most effectively distinguish between MD patients and normal controls.

**Methods:**

Gadolinium-enhanced EH imaging was performed to quantitatively evaluate the extents of hydrops in MD patients and age−/sex-matched normal controls. Four hours after intravenous injection of contrast agent, MRI was performed using a 3-T MR platform fitted with a 32-channel phased-array coil receptor. MR images (10–15 slices) covering an inner ear were 3D-stacked. Analyses of all images that included the vestibule or the cochlea yielded the volumes (in μL) of the endolymphatic and perilymphatic spaces.

**Results:**

For the vestibule, they were significantly greater EH% in ipsilateral (52.4 ± 12.5) than in contralateral MD ears (40.4 ± 8.5, *p* = 0.001) and in ipsilateral MD ears than in control ears (42.4 ± 13.7, *p* = 0.025). For the cochlea, the values were slightly higher EH% in ipsilateral MD ears (49.7 ± 10.4, *p* = 0.061) but did not significantly differ from contralateral (41.3 ± 12.6) or control ears (39.6 ± 18.9, *p* = 0.858). In the MD group, the EH asymmetries were 12.0 ± 10.2% (vestibule) and 8.4 ± 8.6% (cochlea), significantly larger than those of controls.

**Conclusion:**

Compared to conventional semiquantitative grading or quantitative EH% analysis, EH asymmetry may better distinguish MD patients from normal controls. Quantitative hydrops volumetric analysis yields clinically relevant information on inner ear function.

## 1 Introduction

Meniere’s disease (MD) is typically diagnosed based on clinical findings such as aural fullness, tinnitus (1), dizziness, and hearing loss. Before endolymphatic hydrops imaging (EHI) became possible, a diagnosis of certain MD required histopathological evidence (2, 3). However, this was impossible for living individuals; most current guidelines recommend the use of clinical symptoms (4–6). In particular, the 2015 Guideline of the Classification Committee of the Barany Society (CCBS) is based entirely on clinical symptoms, which are preferred to objective tests (5). Recently, magnetic resonance imaging (MRI) of endolymphatic hydrops has made good progress (7, 8). Several studies have shown that EHI well-predicts the pathological side (9–11). Good correlations have been reported between the extent of hydrops and clinical presentations; the former correlates positively with disease duration and the level of hearing loss (9). Imaging techniques such as “hybrid of the reversals of the positive and native images of the perilymph signal” (HYDROPS2) rapidly yield good-quality data (10). The three-dimensional fluid-attenuated inversion recovery (3D-FLAIR) technique can differentiate a variety of dizziness disorders with similar symptoms (7, 11, 12). EHI usefully identifies vestibular and cochlear MD and excludes vestibular migraine (7, 13–16). However, despite such progress, semiquantitative grading of hydrops remains unsatisfactory. A small number of grades is inappropriate; hydrops is not categorical but rather continuous. Bernaerts et al. (17) recently emphasized the greater utility of four-stage vestibular hydrops grading compared to the original Baráth (18) three-stage system. However, one additional grade is not a major advance. In addition, the grading systems usually assume that a single axial slice of the inner ear represents the entire cochlea or vestibule. If endolymphatic distension is rostrocaudal, a single axial slice may not capture the full extent of the hydrops. Some authors have sought to obtain better views of the saccule and utricle by reformatting MR images in the parasagittal plane (19). Saccule-to-utricle ratio inversion (SURI) addresses the inadequate imaging afforded by only axial views. However, SURI evaluates only a single slice of the 3D endolymphatic space. As EHI commonly yields over 10–15 slices of the inner ear, it seems unreasonable to evaluate only one slice. We recently analyzed the inner ear in a volumetric manner; we sought to be holistic (20). A semiquantitative grading system may be adequate if the hydrops location and direction are typical, but in some cases, conventional axial views or reformatted SURI images may fail to accurately reflect the exact extent of hydrops, again because only single slices are evaluated.

In this study, we used gadolinium-enhanced EHI to quantitatively evaluate the extents of hydrops in MD patients and age−/sex-matched normal controls. We verify the utility of 3D measurements by deriving correlations between the extent of hydrops and standard measures of inner ear function (the caloric test and pure tone audiometry [PTA]). We emphasize the importance of evaluating both ears of MD patients because some normal controls also exhibit hydrops despite the absence of otological symptoms. Our study is unusual in that we used EH asymmetry to distinguish MD patients from normal controls. We also performed 3D volumetric evaluations of each entire inner ear.

## 2 Methods

### 2.1 Patients

We obtained quantitative EHI data on 58 ears, of which 15 were ipsilesional MD ears, 15 contralesional non-MD ears, and the remaining 28 control ears. Fifteen patients were clinically diagnosed with MD (definite [*n* = 11] or probable [*n* = 4]) using the 2015 CCBS criteria (5). Fourteen age- and sex-matched controls [28 control ears = 14 controls] were recruited to compare the extents of hydrops between the two groups. The exclusion criteria were a history of any ear disorder such as acute or chronic otitis media or otosclerosis, seizure, or an organic brain disorder; cardiac pacemaker use; and/or any electronic implant including a cochlear implant (21) or intraocular ferromagnetic materials and particles. At evaluation, all MD patients were stable in that they had not experienced severe dizziness within the last month and no changes in hearing had occurred over the past 2 months. Subject demographics are listed in Table 1.

**Table 1:** Subject demographics.

	Meniere’s disease (*n* = 15)	Healthy controls (*n* = 14)
Sex (male:female)	7:8	7:7
Age (years)	47.2 ± 8.4	46.0 ± 8.87
Diagnosis (definite:probable)	11:4	-
Pathological side (right:left)	4:11	-
Duration of dizziness (min)	44.9 ± 60.2	-
Duration of illness (months)	52.5 ± 65.7	-
Tinnitus (yes:no)	10:5	0:14
Ear fullness (yes:no)	10:5	0:14
Hearing fluctuation (yes:no)	9:6	0:14
Caloric test (canal paresis %)	31.6 ± 33.6	-

Data are expressed as means ± standard deviations.

### 2.2 MRI

Four hours after intravenous injection of contrast agent (Magnevist, Bayer Ltd., Germany), MRI was performed using a 3-T MR platform (3-T Magnetom Tim Trio Scanner; Siemens Medical Solutions, Erlangen, Germany) fitted with a 32-channel phased-array coil receptor (7). The MR protocols were identical to those of a previous study (22). Patients underwent heavily T2-weighted (hT2w) MR cisternography (MRC) to anatomically locate all lymph fluid, followed by hT2w 3D-FLAIR with an inversion time of 2,250 ms to obtain perilymph-positive images (PPIs). A variable flip angle 3D-turbo spin-echo sequence followed; this was the “sampling perfection with application-optimized contrast using different flip angle evolution” (SPACE) protocol. The parameters were repetition time (TR) 4,400 ms; echo time (TE) 546 ms; initial refocusing at a 180° flip angle that rapidly decreased to a constant 120° during the turbo spin-echo refocusing echo train; echo train length 203 with a restorative magnetization pulse (a fast recovery pulse); matrix size 319 × 384; 104 axial slices each 1.0 mm thick covering the entire labyrinth; field of view (FOV) 15 × 18 cm; use of the “generalized auto calibrating partially parallel acquisition” (GRAPPA) parallel imaging technique; acceleration factor 2; number of excitations (NEX) 4; and scan time 6 min 30 s. The hT2W-3D-FLAIR scan parameters for PPI were similar except for application of an inversion pulse for 2,250 ms, a TR of 9,000 ms, an NEX of 4, and a scan time of 15 min 32 s. PPI did not employ a restorative pulse. Both MRC and PPI employed an identical FOV, matrix size, and slice thickness. HYDROPS2 images were generated by subtracting the MRC images multiplied by 0.05 from the PPI images. During subtraction, negative HYDROPS2 values were permitted. Acquisition of the source HYDROPS2 images required 18 min. Each HYDROPS2-Mi2 image was obtained by multiplying the MRC and HYDROPS2 images (22).

### 2.3 Endolymphatic hydrops analysis

**Figure 1:**
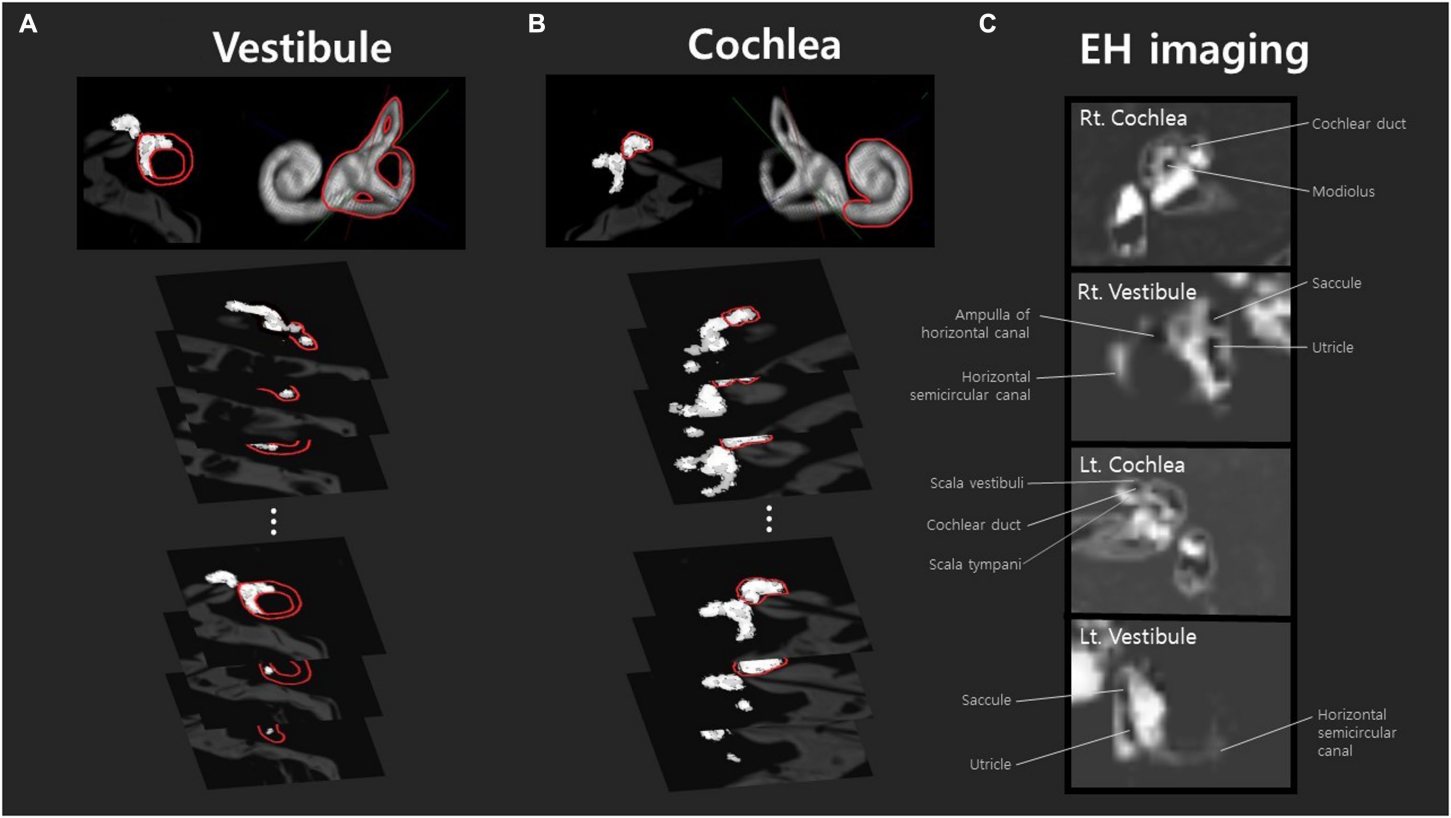
Volumetric analysis of endolymphatic hydrops in the vestibule and cochlea was conducted using MR axial-view images (10–15 slices) sliced at 2 mm intervals. These images were stacked to generate three-dimensional volumetric data. Analyses of all images that include the vestibule **(A)** or cochlea **(B)** yielded the volumes (μL) of the endolymphatic and perilymphatic spaces. Peri- and endolymphatic areas were defined according to our previous literature (20). The volume of each area was calculated by summing the pixel volumes, estimated based on the physical dimensions of the image matrix, slice thickness, and field of view (FOV). Panel **(A)** is an example analysis of the right ear of a patient with mild hydrops, and panel **(B)** is an example analysis of the cochlea with mild hydrops. The images in panel **(C)** are from a subject with mild hydrops in both ears. The cochlea and vestibular canals of both the right and left sides are listed in order, and identifiable structures such as the cochlear duct, saccule, utricle, and ampulla of the horizontal canal can be observed.

Clear demarcations between the perilymph and endolymph were apparent in all 58 ears. When the MRI acquisition parameters or the DICOM viewer window brightness are changed, the extent of hydrops varies. The image acquisition parameters may differ among MRI devices. A threshold technique based on the signal intensities of the HYDROPS2-Mi2 images was utilized to measure the volumes of the endo- and peri-lymphatic spaces which were, respectively segmented using the negative (< −1) and positive (> 5) threshold signal intensities of manually drawn regions of interest (ROI) of the cochlea and vestibule apparent on the MR cisternographs. Although the signal intensity of any bony structure in a HYDROPS2-Mi2 ROI was set to zero, the value for any bone in the cochlea and vestibule might be non-zero. The cutoffs (−1 and 5) were chosen to minimize any overestimations of volumes within the boundaries of the cochlea and vestibule. The details have previously been described (20). In brief, all MR images (10–15 slices) covering an inner ear were 3D-stacked (Figure 1). Analyses of all images that included the vestibule or the cochlea yielded the volumes (in μL) of the endolymphatic and perilymphatic spaces. The quantitative volumetric EH% = endolymph volume (μL)/(endolymph+perilymph volume [μL]) was calculated by the image analysis software. The EH% values of MD and non-MD ears were subtracted to obtain the binaural EH asymmetry as (ipsilateral MD ear EH%) – (contralateral ear EH%). For normal controls, the binaural EH asymmetry was (right ear EH%) – (minus left ear EH%).

### 2.4 Caloric test and PTA

The bithermal caloric test employed cool (30°C) and warm (44°C) water (Variotherm Plus; Atmos, Allentown, PA, USA). Nystagmus was quantified using a VisualEyes system (Micromedical, Chatham, IL, USA). Unilateral vestibular hypofunction was diagnosed when the canal paresis was ≥25%. PTA employed an AD229b diagnostic audiometer (Interacoustics, Assen, Denmark) and a soundproof booth. PTA data were acquired at 0.25, 0.5, 1, 2, 4 and 8 kHz. The four-tone average (mean of 0.5, 1, 2, and 4 kHz) was used for correlation analysis.

### 2.5 Statistical analysis

Continuous variables are expressed as means ± standard deviations (SDs). All comparisons employed SPSS version 25.0 software (SPSS Inc., Chicago, IL, USA). The Kruskal-Wallis and Mann–Whitney *U*-tests were used as appropriate, to compare the extents of endolymphatic hydrops between MD patients and controls. Spearman correlations were derived. Receiver operating characteristic (ROC) curve and the area under the ROC curve (AUC) were used to determine the outcome measure with higher sensitivity and specificity. *p*-values <0.05 were considered statistically significant.

**Figure 2:**
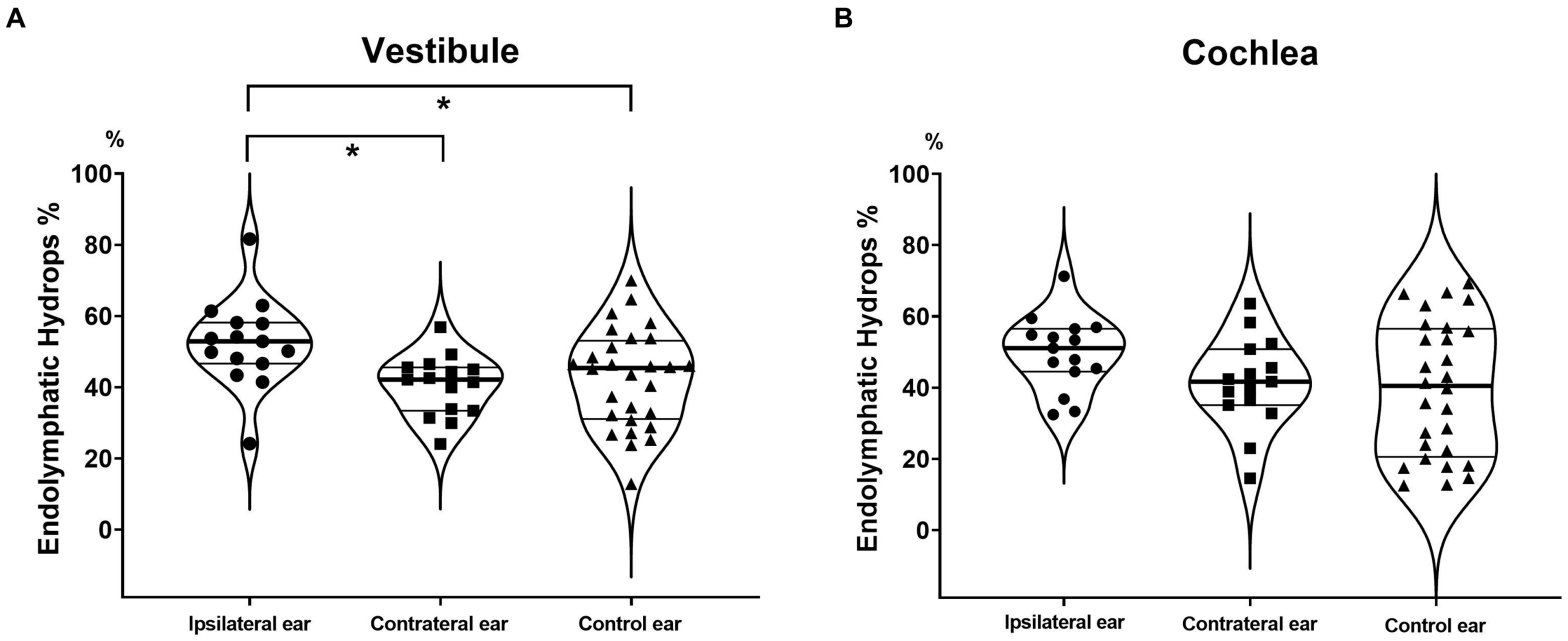
Endolymphatic hydrops percentages (EH% values) of the vestibule and cochlea. The vestibular EH% was significantly greater in ipsilateral MD ears than in contralateral and control ears **(A)**. A similar tendency was evident for the cochlea, but the difference did not attain statistical significance **(B)**.

## 3 Results

### 3.1 Demographics

The distributions of sex (male:female = 7:8), age (47.2 ± 8.4 years) and duration of illness (44.9 ± 60.2 months) in MD patients were similar to those of previous reports (Table 1) (20) and the sex balance (male:female = 7:7) and age (46.0 ± 8.9 years) of the control group were almost identical to those of MD patients. No control subject had a history of dizziness or hearing loss. The hearing thresholds were significantly higher in ipsilateral MD ears than in contralateral ears and healthy control ears (Table 2). The hearing thresholds of the contralateral MD and control ears did not differ.

**Table 2:** PTA data.

Frequency	Ipsilateral MD ears (*n* = 15)	Contralateral ears (*n* = 15)	Control ears (*n* = 28)	*p*-value
250 Hz	52.4 ± 25.1	11.1 ± 7.2*	10.7 ± 7.9*	< 0.001
500 Hz	54.5 ± 26.6	13.9 ± 7.9*	14.0 ± 8.3*	< 0.001
1,000 Hz	53.2 ± 25.9	11.8 ± 8.4*	11.4 ± 9.8*	< 0.001
2,000 Hz	49.2 ± 24.1	13.4 ± 9.9*	13.8 ± 10.3*	< 0.001
4,000 Hz	54.5 ± 23.3	21.3 ± 14.9*	23.0 ± 14.2*	< 0.001
8,000 Hz	64.5 ± 15.4	26.6 ± 23.1*	28.9 ± 24.8*	0.002

Data are presented as means ± standard deviations.

### 3.2 Eh%

For the vestibule, they were significantly greater EH% in ipsilateral (52.4 ± 12.5) than in contralateral MD ears (40.4 ± 8.5, *p* = 0.001) and in ipsilateral MD ears than in control ears (42.4 ± 13.7, *p* = 0.025) (Figure 2A). For the cochlea, the values were slightly higher EH% in ipsilateral MD ears (49.7 ± 10.4, *p* = 0.061) but did not significantly differ from contralateral (41.3 ± 12.6) or control ears (39.6 ± 18.9, *p* = 0.858) (Figure 2B).

### 3.3 EH asymmetry

**Figure 3:**
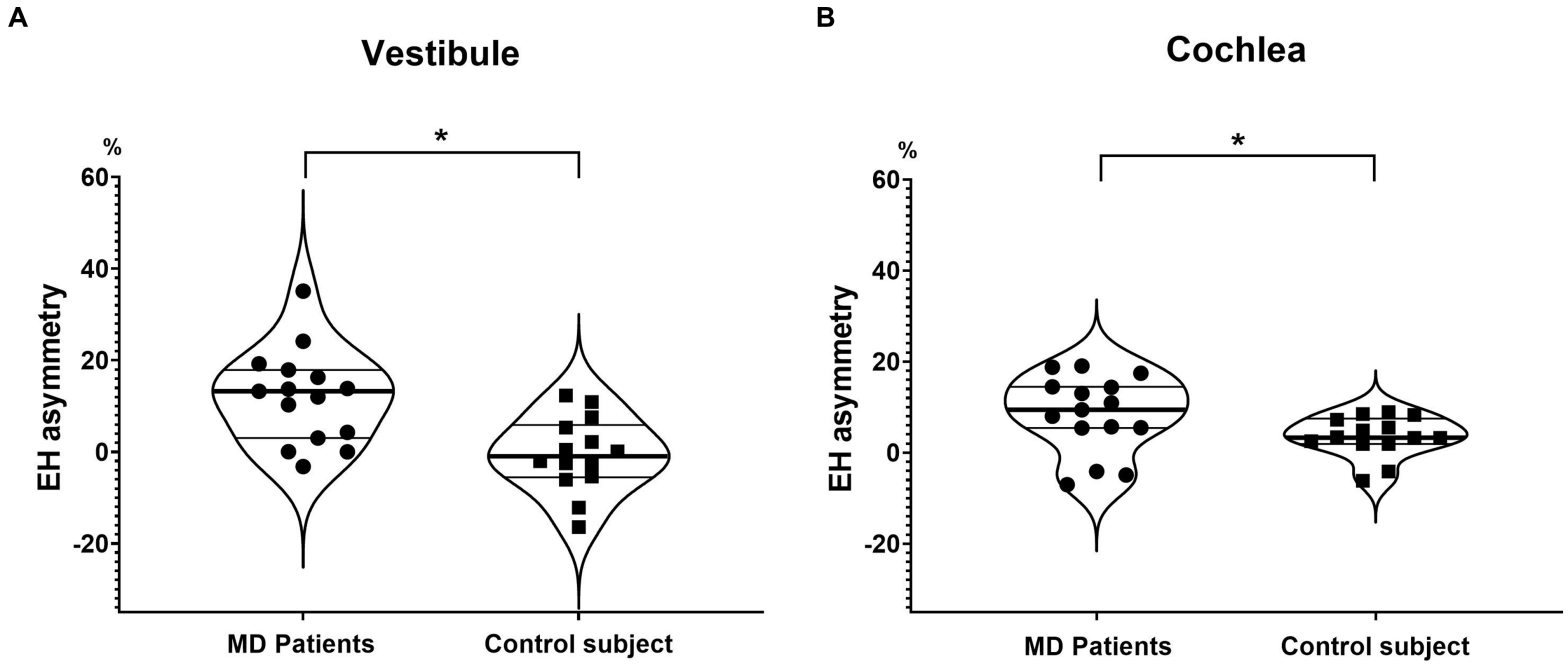
Endolymphatic hydrops asymmetry (EH asymmetry) of the vestibule and cochlea. EH asymmetry of both the vestibule **(A)** and cochlea **(B)** was significantly greater in MD patients than in controls.

In the MD group, the EH asymmetries were 12.0 ± 10.2% (vestibule) and 8.4 ± 8.6% (cochlea), significantly larger than those of controls (Figure 3).

### 3.4 ROC analysis

**Figure 4:**
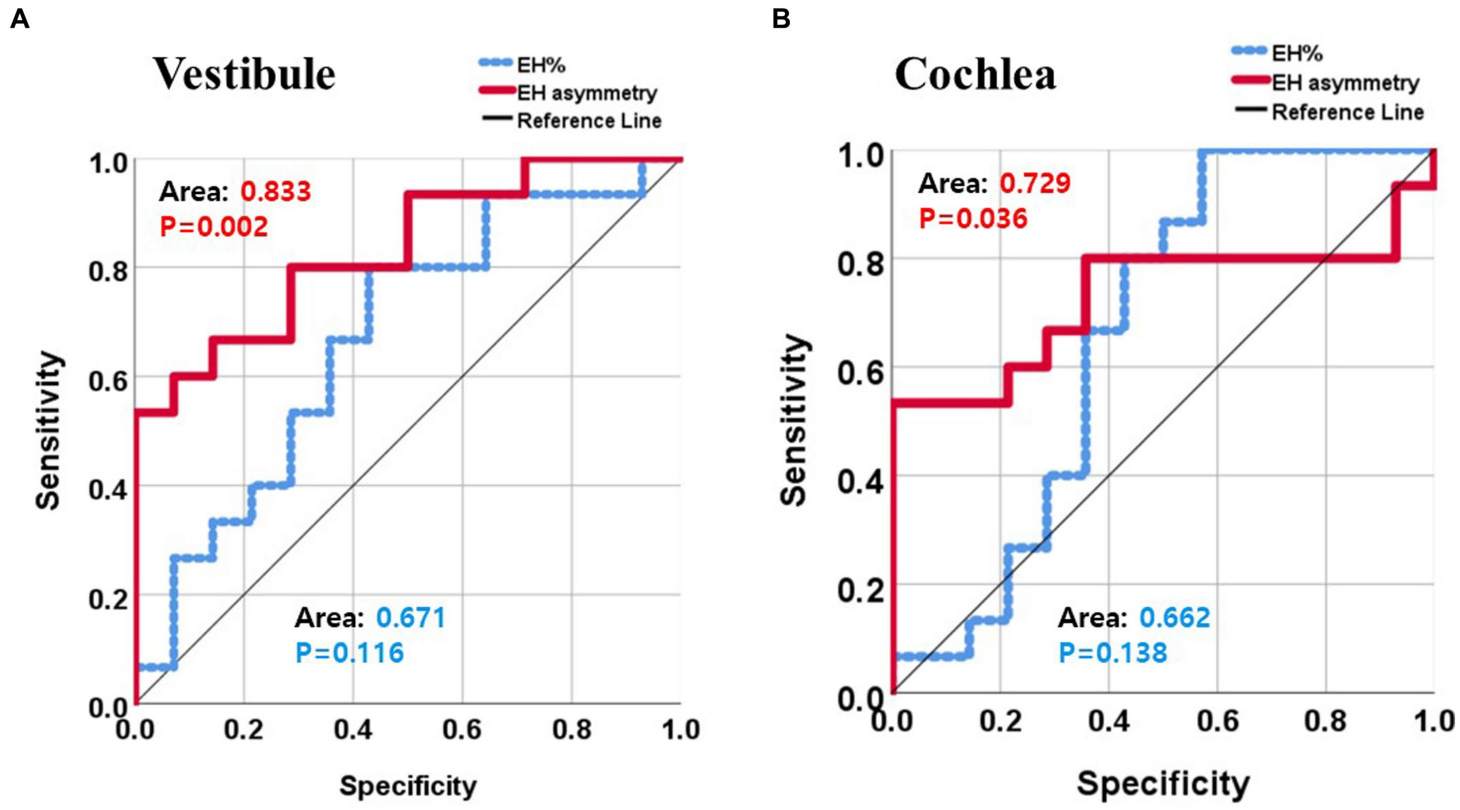
Receiver operator curve analysis distinguishing MD by the endolymphatic hydrops percentage (EH%) and EH asymmetry. EH asymmetry exhibited a higher sensitivity, specificity, and area under the curve than did the EH% in terms of MD diagnosis (**A** and **B**).

When the ROC analysis was performed, EH asymmetry of bilateral ears was superior to EH% on the pathologic ear in distinguishing MD patients from controls. That is, the AUC was 0.833 and 0.671 in the vestibule by EH asymmetry and EH%, respectively (Figure 4A). AUC in the cochlea was 0.729 and 0.662 by EH asymmetry and EH%, respectively (Figure 4B).

### 3.5 Clinical relevance of EH imaging

**Figure 5:**
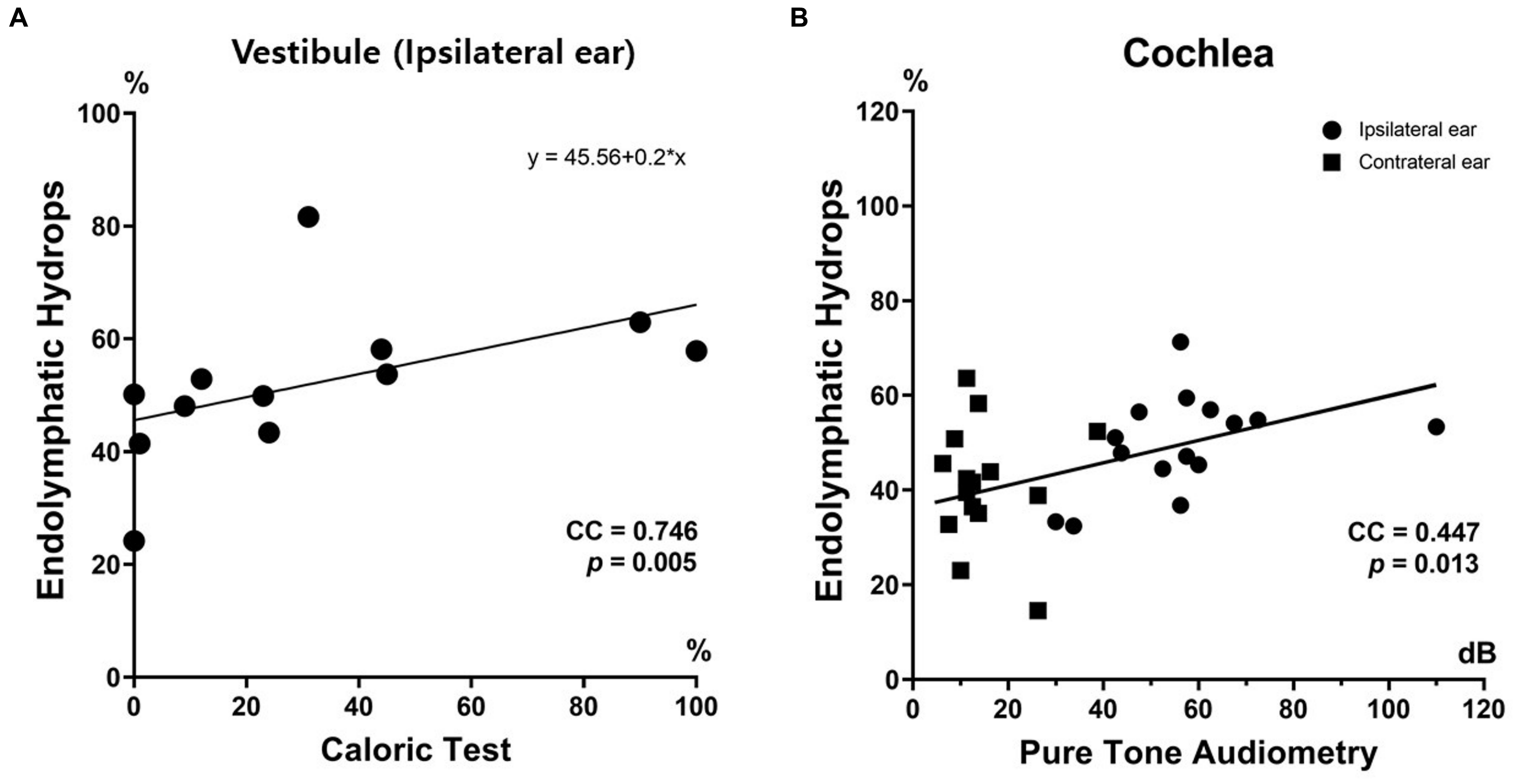
Clinical relevance of endolymphatic hydops imaging. The endolymphatic hydrops percentage (EH%) correlated well with inner ear function. The vestibular EH% of ipsilateral MD ears significantly correlated with the extent of canal paresis (%) revealed by the caloric test **(A)**. The cochlear EH% significantly correlated with the PTA hearing threshold **(B)**.

The correlation between the vestibular EH% of the ipsilateral ear and the caloric CP was significant (Figure 5). Thus, the caloric CP significantly increased as the vestibular EH% of the ipsilateral ear increased (*r* = 0.746; *p* = 0.005). At CP > 25%, the vestibular EH% always exceeded 50%. Conversely, when it was below 50%, the caloric test was normal (with one exception). The vestibular EH% of the ipsilateral ear predicted abnormal caloric CPs in 91.7% of all subjects (*p* < 0.001). In addition, a good correlation was apparent between the EH% and the PTA hearing threshold. That threshold significantly increased as the cochlear EH% increased (correlation coefficient [CC] = 0.447; *p* = 0.013).

### 3.6 Correlations between vestibular and cochlear data

**Figure 6:**
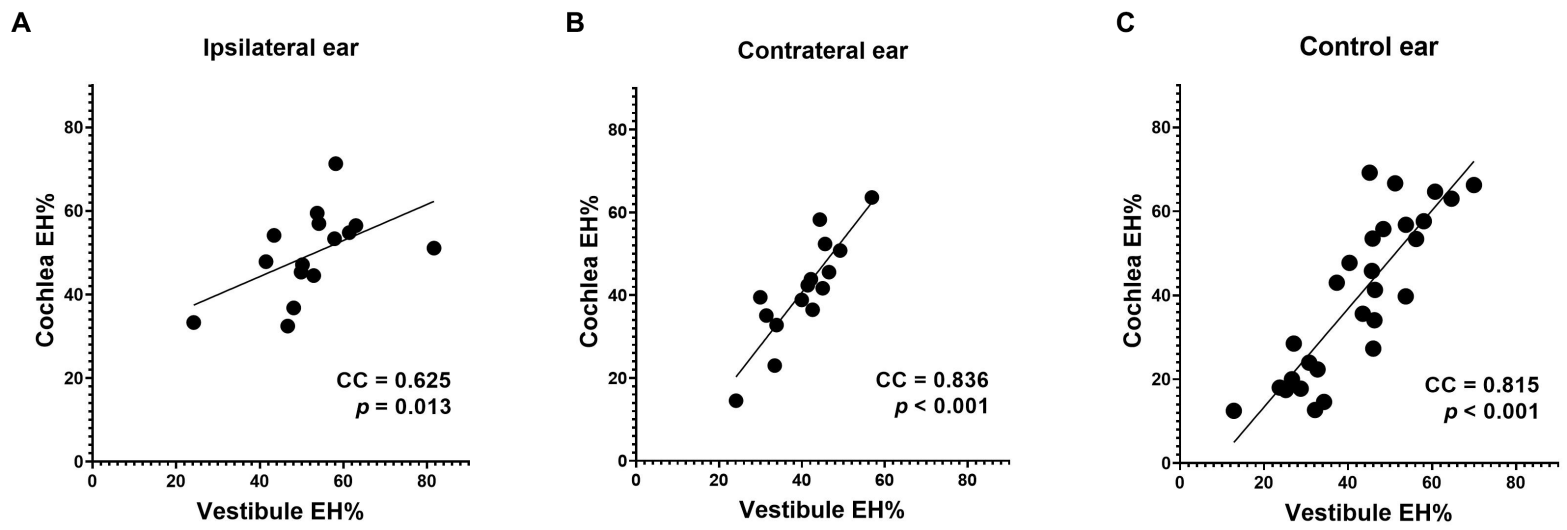
Correlations between the vestibular and cochlear data. Good correlations were apparent between the cochlear and vestibular EH% values of all three groups (**A**, **B** and **C**).

The EH% correlations between the vestibule and cochlea were very high for all three groups (see Figure 6 for data).

## 4 Discussion

We found that EH asymmetry usefully distinguished MD patients from normal controls. The EH% has been widely used to quantitatively measure the extent of hydrops. High EH% values have been considered indicative of pathological hydrops, with low values being normal. However, it has become apparent that normal controls exhibit some hydrops; a high EH% may not always indicate a pathological condition such as MD. The key distinction is the hydrops symmetry; control subjects are symmetrical but MD patients exhibit a unilateral high EH%. EH asymmetry effectively differentiates MD patients from normal controls. In our study, the sensitivity, specificity, and area under the curve (AUC) of EH asymmetry were better than those of conventional EH%. This is akin to the interpretation of caloric or vestibular evoked myogenic potential (VEMP) tests, where greater emphasis is placed on asymmetry between the two ears rather than the absolute values; the absolute responses of single ears may vary widely. Similarly, we maintain that the EH% exhibits a wide range of normal variation, and that the use of EH asymmetry rather than the absolute EH% is thus a reasonable solution to the diagnostic problem. Few controlled studies on volumetric EHI analyses have appeared; most have evaluated the extents of hydrops only in patients with dizziness and/or hearing loss. This is because it is tedious to perform EH MRI of normal controls; the complex imaging requires 4 h and is expensive. The use of the contralateral ear as a control may be both practical and viable. A high EH% in the ipsilateral MD ear and a low EH% in the contralateral ear has consistently been demonstrated in many studies (8, 10, 11, 23), including ours. However, we found that a high EH% alone does not necessarily indicate MD. We enrolled normal controls; all exhibited symmetrically high EH% values.

The sensitivity and specificity of EHI in terms of MD diagnosis remain controversial. Some studies have reported sensitivities of 100% (24); another have reported a range from 73.2 to 94.2% (21). Specificity is more challenging; this depends on the prevalence of hydrops in normal controls, which is about 10% (25). In extreme cases, the incidences of hydrops may be as high as 66% in those with other otological conditions and 90% in healthy volunteers (26). In such a case, EHI poorly identifies normal subjects; the specificity is only 10% for the vestibule and 33% for the cochlea (16). It may be that such a high incidence of hydrops in normal controls reflects a limitation of MRI or EHI. However, histopathological studies have also reported hydrops in subjects lacking MD. One meta-analysis of human data (53 articles, 3,707 temporal bones, and 276 patients) reported hydrops in 105 cases lacking any MD history (27), thus in about 6.4% of all non-MD samples. In general, it appears that both EHI and conventional methods exhibit high sensitivity but low specificity; the latter is in part attributable to the fact that some individuals exhibit a high EH% but no ear symptoms.

Despite such controversies, EHI is now a common clinically relevant tool. Recent studies have used EHI to diagnose MD in more than 300 clinical datasets (7, 8). Commencing in the early 2010s, many works have validated the utility of EHI (28–30) and have used it in clinical settings (7, 31). Quantitative EHI studies have reported meaningful results; MD ears exhibit high EH% values (31, 32). Naganawa et al. reported that 8 of 10 MD patients exhibited high vestibular EH% values in affected ears (31). In another study, 22 of 24 patients (91.7%) with MD of more than 2 years in duration exhibited greater enlargements than others (32). Many studies have reported that EHI is effective; such positive results have generally been reproducible (7, 13, 31). We also found a significant difference in the extents of hydrops between ipsilateral MD and contralateral ears, in line with previous reports. We thus provide additional evidence for the clinical utility of EHI.

Many published studies have lacked normal control groups, limiting our understanding of the clinical relevance of EHI (7, 20, 25). Hydrops is present in about 10% of healthy controls (25); it may not be a pathognomonic sign of a specific disorder. In addition,. The contralateral nonpathological ear is an imperfect control; this is not the ear of a person lacking any inner ear disorder. A normal control group evaluated using the same scanner and acquisition parameters in the same institute is needed. Otherwise, it is difficult to determine if an EHI finding is clinically relevant or pathognomonic. In this study we found that “EH asymmetry” is superior to the “degree of EH on the pathological ear” in distinguishing MD patients from controls. Until now, all EHI studies have focused on the degree of EH in each ear separately. The degree of EH (EH%) has been widely used to represent the severity of MD, but we have found that the sensitivity and specificity of EH% are not adequate, as normal control subjects may also exhibit symmetric EH. We are suggesting a reasonable solution to this diagnostic problem by providing evidence that “EH asymmetry” excels in terms of sensitivity, specificity, and AUC of the ROC curve. Despite similar looking hydrops in MRI, hydrops in normal controls may be different from hydrops in MD patients. That is, while EH in normal controls is a systemic condition affecting both ears, EH in MD patient is a localized condition that is concentrated only on the pathologic ear.

Hydrops MRI reveals EH and can be used to diagnose MD (8). However, some limitations remain. First, the resolution of MRI is low compared to the size of the inner ear. The voxel size of a typical MRI scan is 0.47 × 0.47 × 1.00 mm^3^ (33) and the inner ear volume is usually 180–300 mm^3^ (34, 35). Thus, the use of only 1,000–1,300 voxels to image the entire inner ear does not reveal small details well. The boundary between endolymph and perilymph may be blurred, associated with errors during quantitative analyses. Second, endolymphatic hydrops may be shared by various inner ear disorders, and thus is not pathognomonic of MD. Although the incidences are low, patients with vestibular neuritis and vestibular migraine may also exhibit hydrops (14, 36). Thus, hydrops in the vestibule or cochlea may be attributable to a condition other than MD (37). Third, EHI does not directly reflect the endolymphatic pressure. MD patients often report aural fullness prior to the development of vertigo and hearing loss. A pressure build-up may distend the endolymphatic space, associated with a high EH% in EHI. The relationship between endolymphatic pressure and volume may not be linear. It is likely that the dilated endolymphatic volume does not return to normal after recurrent attacks even when the endolymphatic pressure has normalized; a large-volume low-pressure state develops. Therefore, a high EH% (indicating a large volume) apparent in MRI may reflect a history of high-pressure episodes, not necessarily a high pressure at the time of imaging.

Our study had certain limitations. First, although most patients had definite MD, a small number had been diagnosed with probable MD. However, the patient demographics and principal outcomes were similar to those of previous studies (18). Second, the number of subjects was low. However, the statistical power was adequate to demonstrate a significant difference between the groups. A larger number of subjects might have rendered our conclusions even stronger.

## 5 Conclusion

Compared to conventional semiquantitative grading or quantitative EH% analysis, EH asymmetry may better distinguish MD patients from normal controls: higher sensitivity, specificity, and AUC. Quantitative hydrops volumetric analysis yields clinically relevant information on inner ear function.

## Data availability statement

The original contributions presented in the study are included in the article/Supplementary material, further inquiries can be directed to the corresponding authors.

## Ethics statement

The studies involving humans were approved by institutional review board at Seoul National University Hospital (H-1806-047-950): Hyun-hoon Jung, Yun-jun Kim, Cheol Kyu Yoo, Jigon Ryu, Hwal Lee, Biryong Joe, Kijeong Cheon, and Yongmin Ahn. The studies were conducted in accordance with the local legislation and institutional requirements. The participants provided their written informed consent to participate in this study. Written informed consent was obtained from the individual(s) for the publication of any potentially identifiable images or data included in this article.

## Author contributions

T-SN: Formal analysis, Methodology, Software, Writing – original draft. MP: Conceptualization, Supervision, Writing – review & editing. JL: Investigation, Supervision, Writing – review & editing. SO: Investigation, Supervision, Writing – review & editing. J-hK: Investigation, Supervision, Writing – review & editing. IS: Conceptualization, Formal analysis, Methodology, Software, Writing – review & editing. M-WS: Conceptualization, Data curation, Methodology, Writing – original draft, Writing – review & editing.
