# Supplementary material for: Endolymphatic hydrops asymmetry distinguishes patients with Meniere’s disease from normal controls with high sensitivity and specificity
Source: Front Neurol. 2023 Dec 21;14:1280616. doi: 10.3389/fneur.2023.1280616 (PMC10768198; doi:10.3389/fneur.2023.1280616)
Supplement: Supplementary file 1 [file Image_1.pdf]

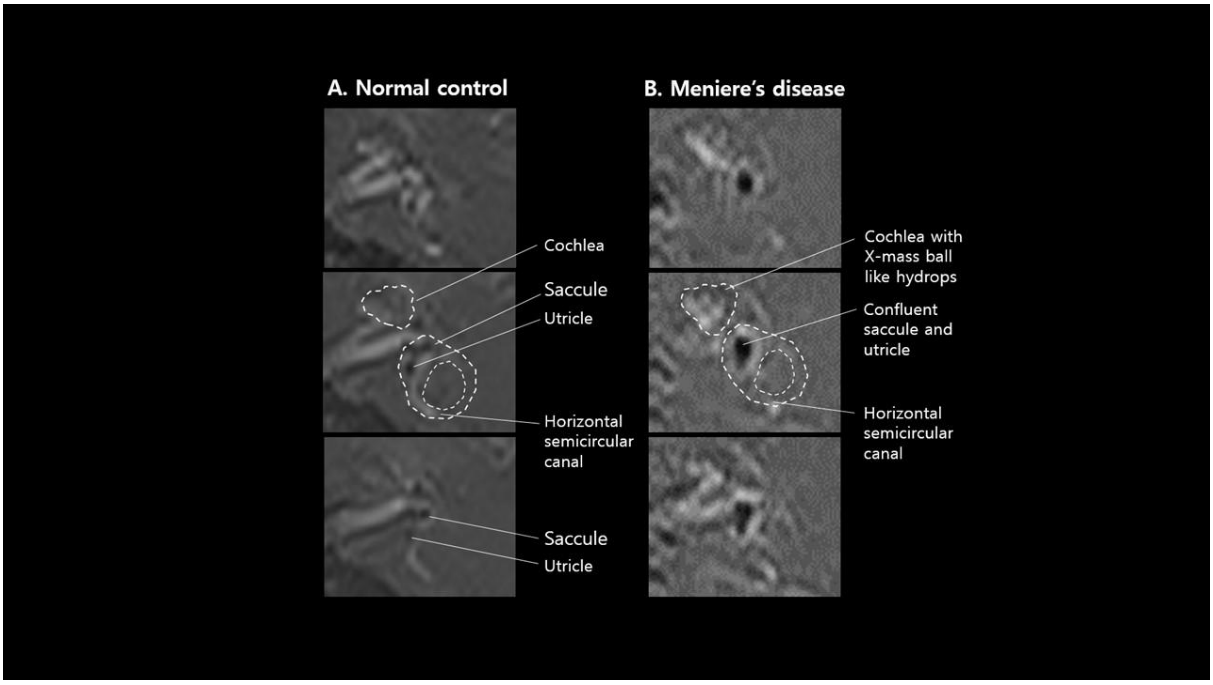

Supplementary Figure 1: A comparison between the normal control and Meniere's disease in terms of hydrops degree.

The cochlea and vestibule are depicted using the improved 3D inversion-recovery (3D-IR) turbo spin echo with real reconstruction. In the normal control ear (A), the saccule and utricle are distinctly demarcated. In contrast, in Meniere's disease (B), they merge and appear enlarged. The expanded endolymphatic duct in the cochlea resembles Christmas tree balls in Meniere's disease (classified as grade 1 hydrops according to the Bernaerts classification).
