# Supplementary material for: Endolymphatic hydrops asymmetry distinguishes patients with Meniere’s disease from normal controls with high sensitivity and specificity
Source: Front Neurol. 2023 Dec 21;14:1280616. doi: 10.3389/fneur.2023.1280616 (PMC10768198; doi:10.3389/fneur.2023.1280616)
Supplement: Supplementary file 2 [file Image_2.pdf]

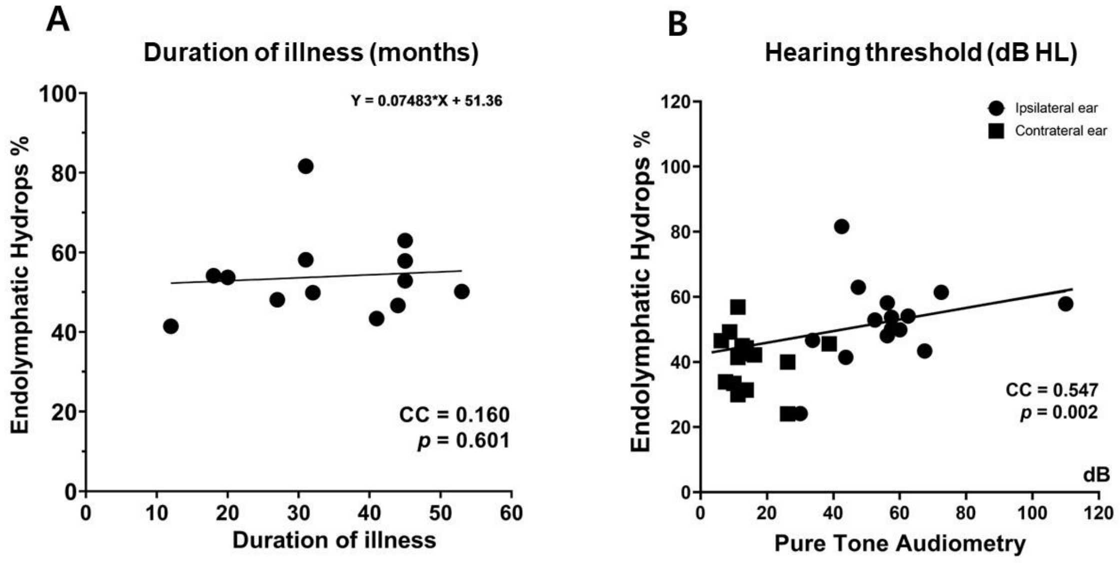

Supplementary Figure 2: Clinical Relevance of Endolymphatic Hydrops Imaging.

The endolymphatic hydrops percentage (EH%) of the vestibule did not show a significant correlation with the duration of illness. The degree of EH was rather stationary (50-60%), regardless of the duration of illness (correlation coefficient = 0.160, p = 0.601) (A). Meanwhile, EH% of the vestibule significantly correlated with the hearing threshold (correlation coefficient = 0.547, p = 0.002) (B). Although direct causality needs further investigation, it seems that the degree of EH is related to the gradual progression of hearing loss.
